# Supplementary material for: Genome-wide bioinformatics analysis of human protease capacity for proteolytic cleavage of the SARS-CoV-2 spike glycoprotein
Source: Microbiol Spectr. 2024 Jan 8;12(2):e03530-23. doi: 10.1128/spectrum.03530-23 (PMC10846095; doi:10.1128/spectrum.03530-23)
Supplement: Supplemental Figures — Figures S1 to S7. [file spectrum.03530-23-s0002.pdf]

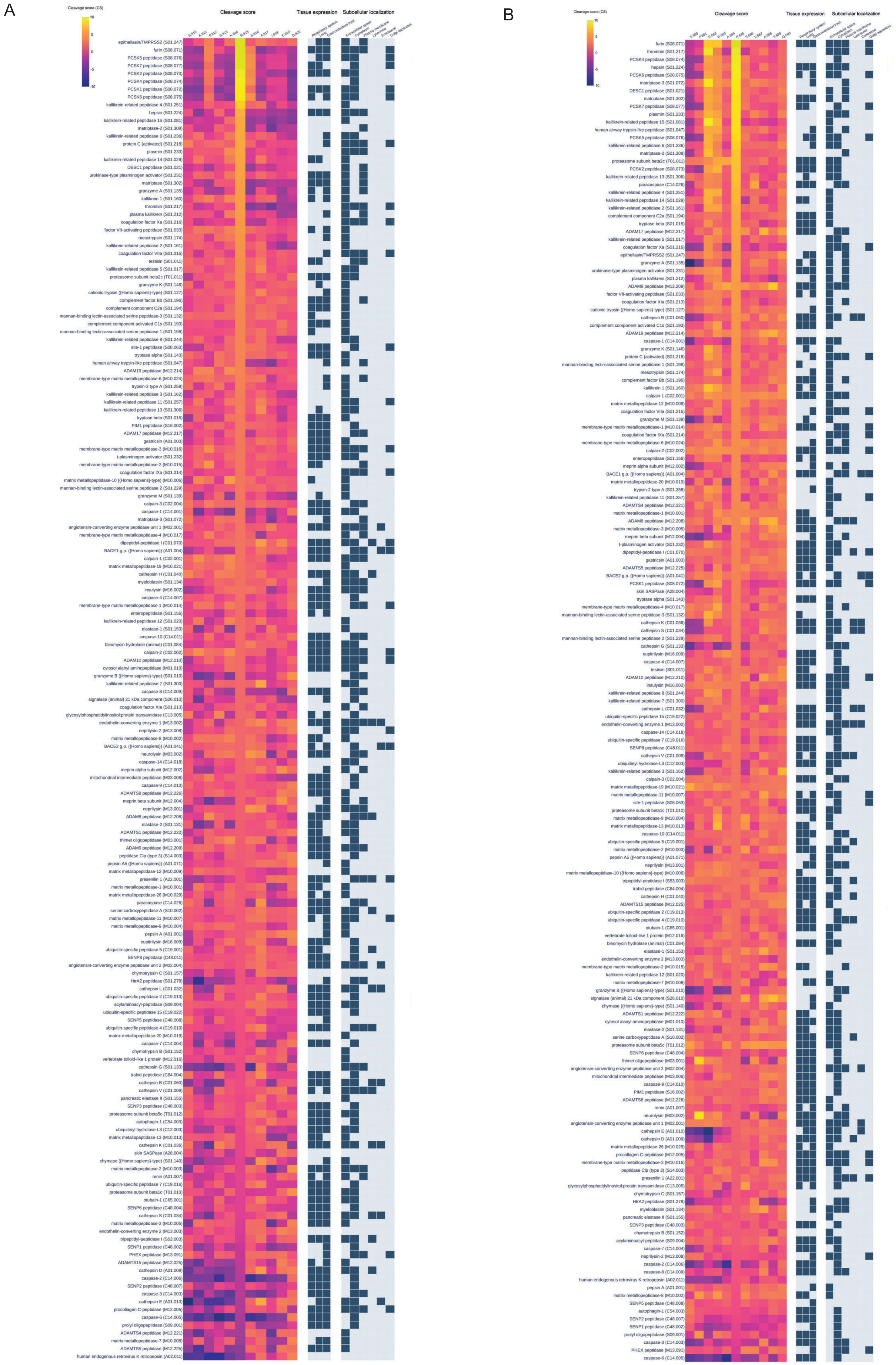

Figure S1. (a) Cleavage scores at the R815 (a) and R685 (b) positions, protease cellular localization, and protease tissue expression for the 169 proteases with modeled protease sequence specificity.

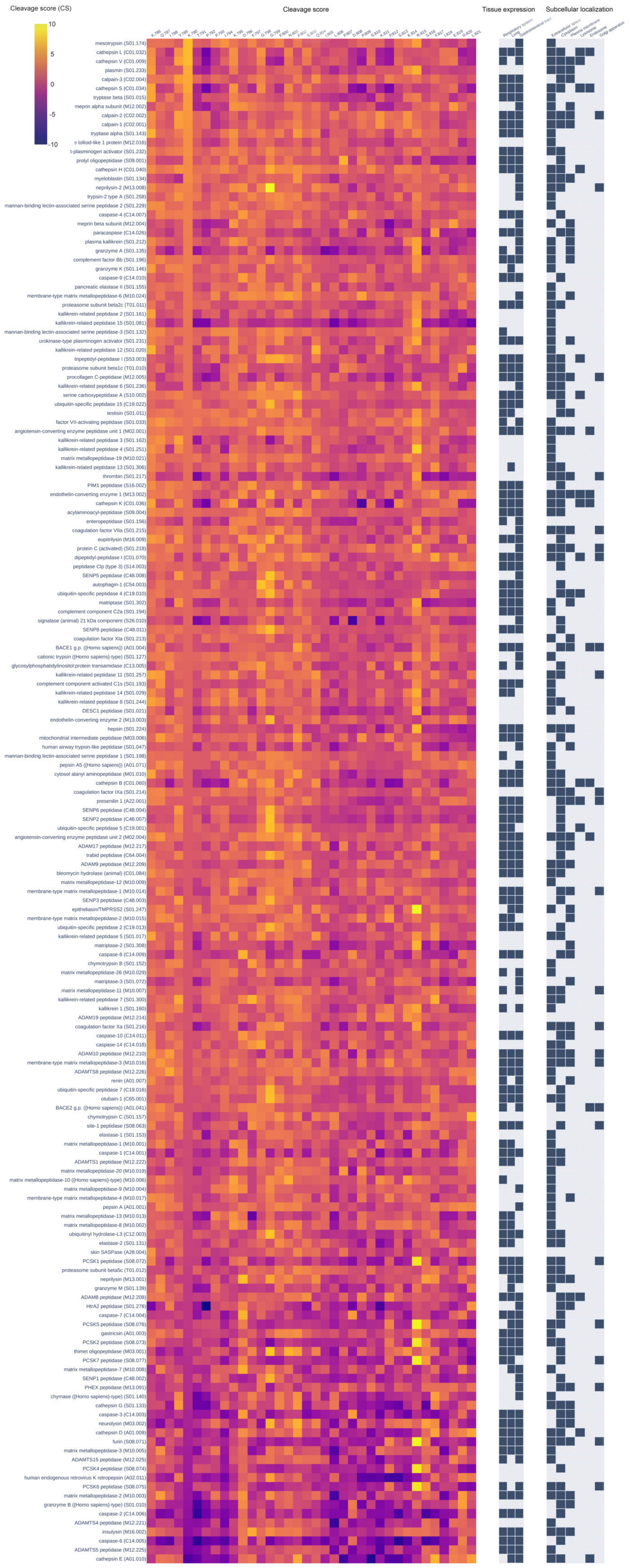

Figure S2. Cleavage scores at the K790 position, protease cellular localization, and protease tissue expression for the 169 proteases with modeled protease sequence specificity.

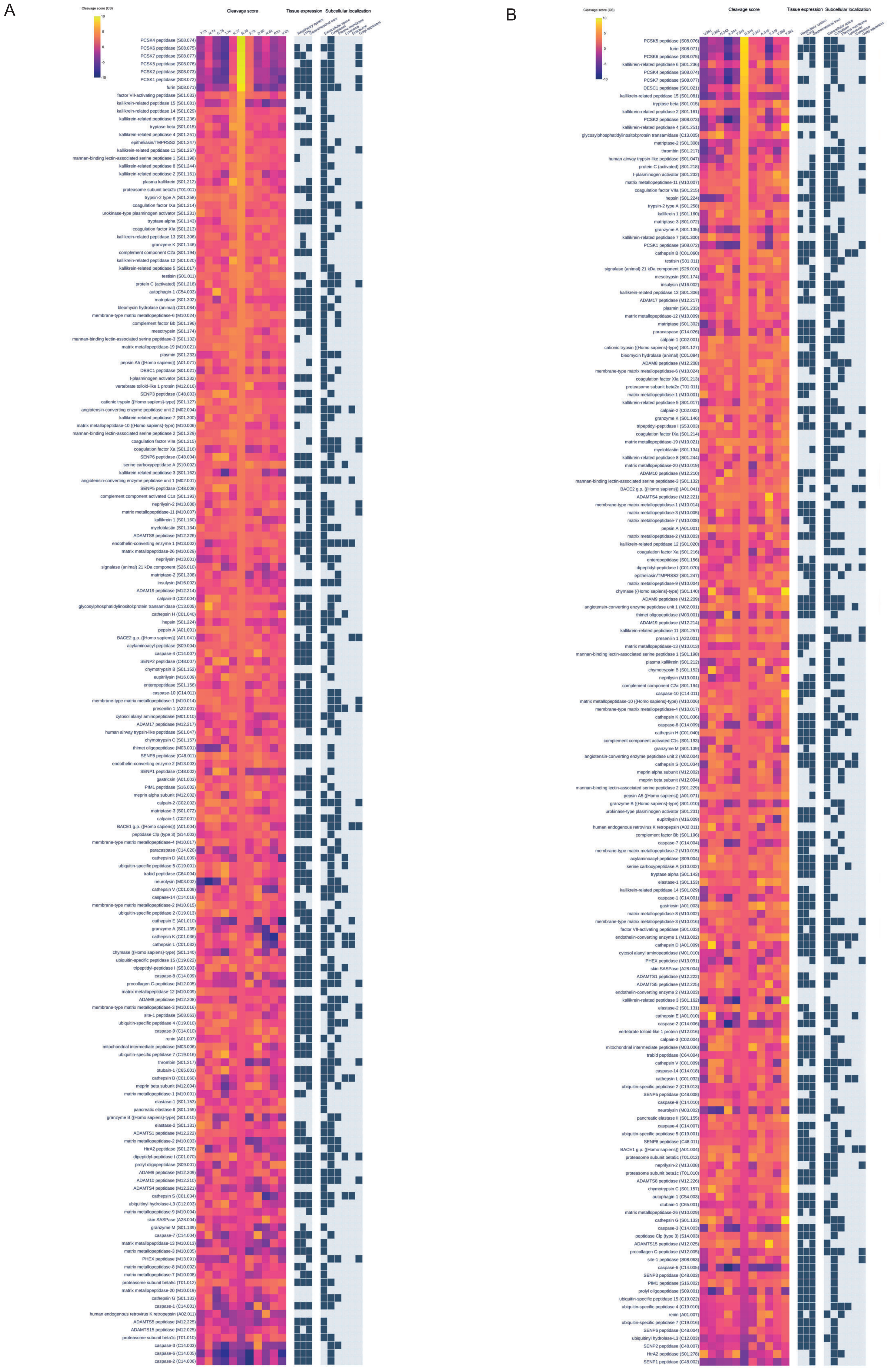

Figure S3. (a) Cleavage scores at the R78 (a) and R346 (b) positions, protease cellular localization, and protease tissue expression for the 169 proteases with modeled protease sequence specificity.

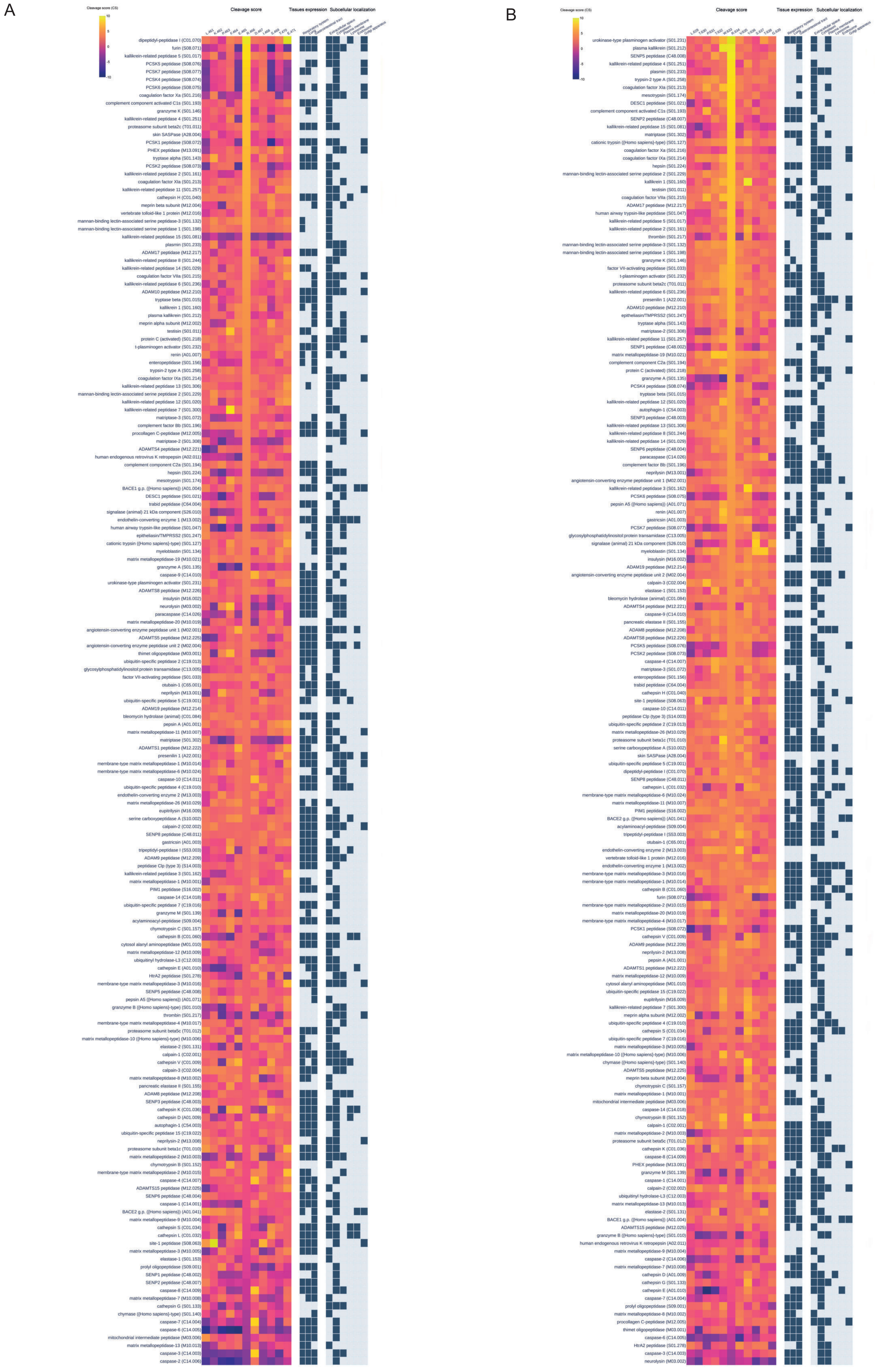

Figure S4. (a) Cleavage scores at the R466 (a) and R634 (b) positions, protease cellular localization, and protease tissue expression for the 169 proteases with modeled protease sequence specificity.

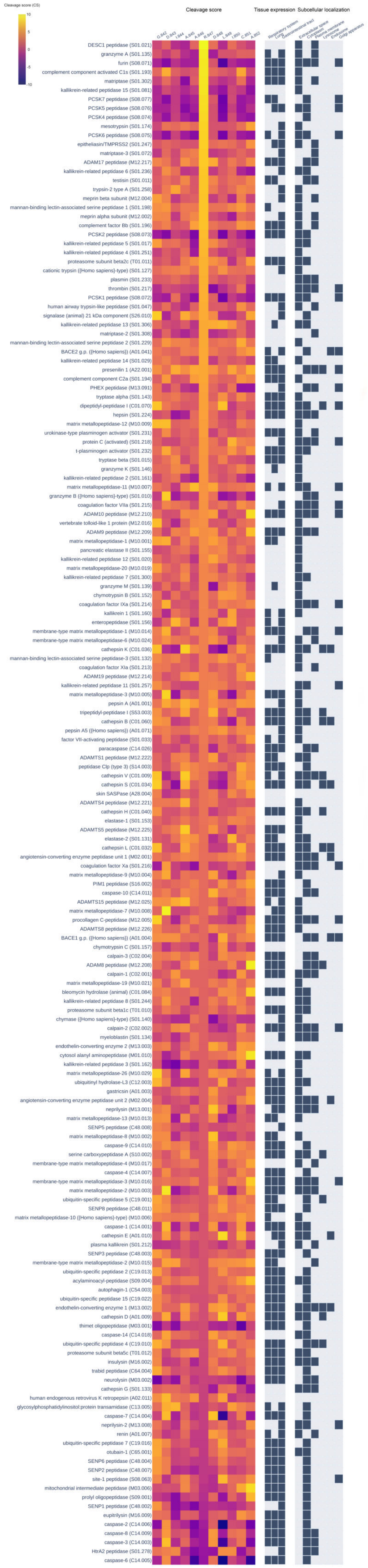

Figure S5. Cleavage scores at the R847 position, protease cellular localization, and protease tissue expression for the 169 proteases with modeled protease sequence specificity.

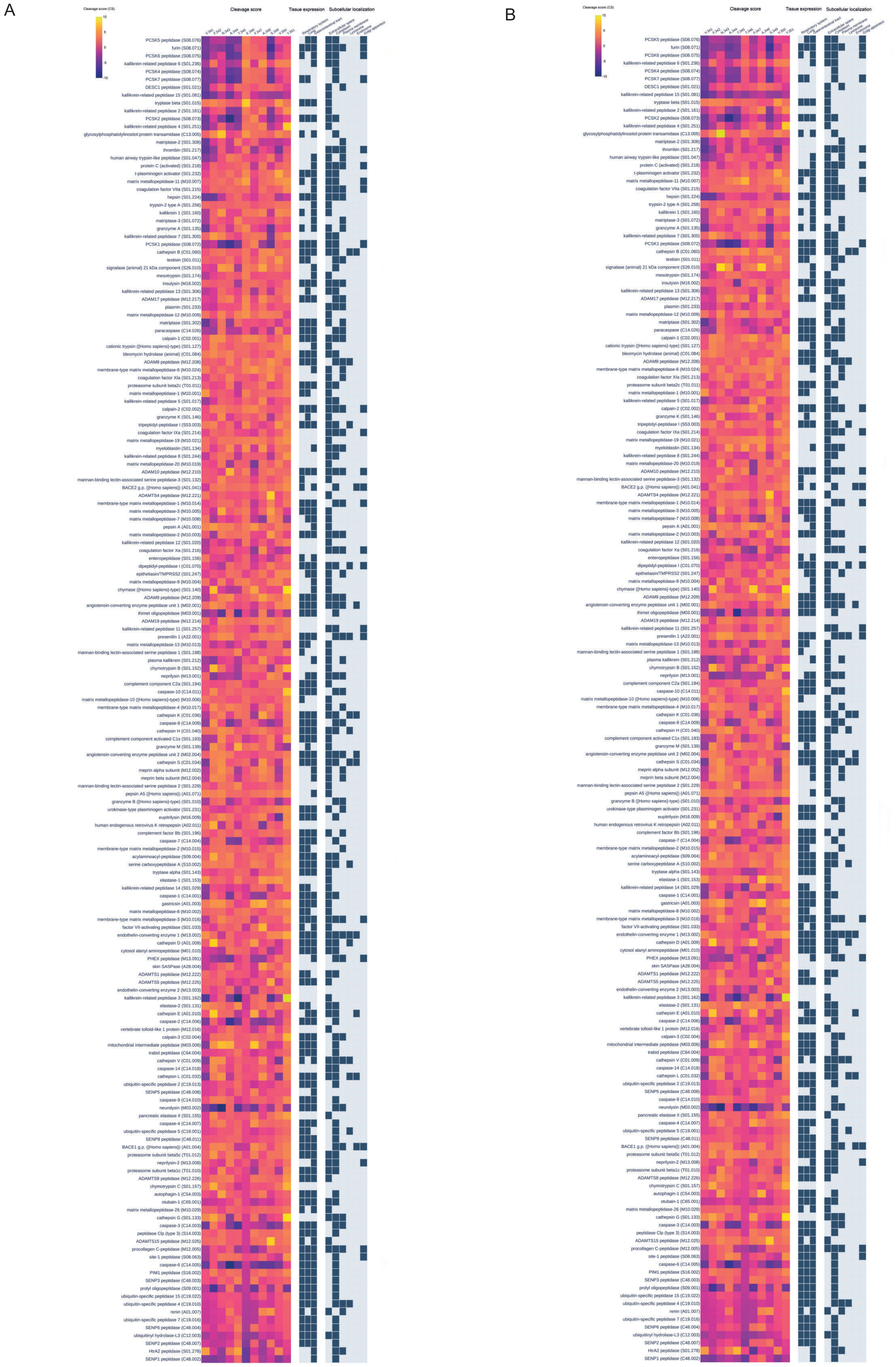

Figure S6. (a) Cleavage scores at the R346 position for R346K (a) and R346T (b) mutations, protease cellular localization, and protease tissue expression for the 169 proteases with modeled protease sequence specificity.

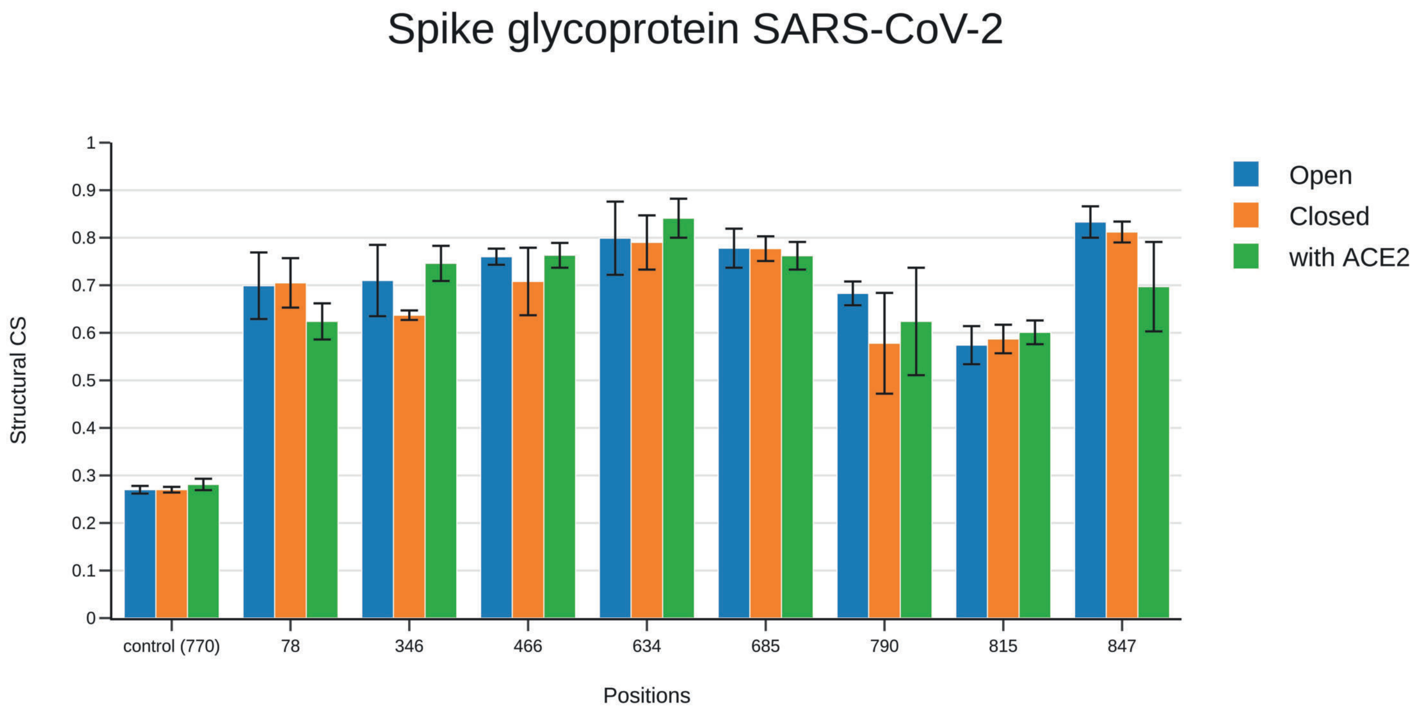

Figure S7. Structural estimation of susceptibility to proteolysis for known and potential cleavage sites of the spike glycoprotein based on 3D structures from different SARS-CoV-2 variants (Alpha, Beta, Delta, Kappa, Omicron).
